# Development and validation of a screening instrument for borderline personality disorder (SI-Bord) for use among university students

**DOI:** 10.1186/s12888-020-02807-6

**Published:** 2020-08-17

**Authors:** Trustsavin Lohanan, Thanakorn Leesawat, Tinakon Wongpakaran, Nahathai Wongpakaran, Nuntaporn Karawekpanyawong, Awirut Oon-Arom, Pimolpun Kuntawong

**Affiliations:** 1 Faculty of Medicine, Chiang Mai University, Chiang Mai, Thailand; 2 Department of Psychiatry, Faculty of Medicine, Chiang Mai University, 110 Intawaroros Rd., T. Sriphum, A. Muang, Chiang Mai, 50200 Thailand

**Keywords:** Borderline personality disorder, Screening, Validation, Instrument, Undergraduate

## Abstract

**Background:**

The screening instrument for borderline personality disorder (SI-Bord) consists of a 5-item self-reported questionnaire on the key features of BPD from the *DSM-5* using a 5-point Likert scale. This study investigated its validity and reliability in screening for BPD in university students.

**Methods:**

A cross-sectional study was conducted on a sample of university students in Thailand between November and December 2019. An online assessment gathered demographic data and results from the SI-Bord, the Perceived Stress Scale-10 (PSS-10) and the Patient Health Questionnaire-9 (PHQ-9). Participants whose SI-Bord scores were ≥ 1 were randomly selected to be interviewed and assessed for a BPD diagnosis by four psychiatrists using the Structured Clinical Interview for DSM-IV Axis II Personality Disorders (SCID-II) as a reference point. An intraclass correlation coefficient (ICC) of 0.925 (95% CI, 0.805–0.979) ensured inter-rater reliability between the four psychiatrists. The diagnostic sensitivity and specificity of the SI-Bord, as compared to that of the SCID-II, were determined to indicate the cut-off score. The Receiver Operating Characteristics (ROC) was analyzed to evaluate its diagnostic accuracy.

**Results:**

The study included 342 students aged 18–25 years (the mean age was 20.25 ± 1.4 years), 80.4% of whom were female. Among the 68 participants selected for an online interview, 16 were diagnosed with BPD. The cut-off score of the SI-Bord was > 9, as suggested by the Youden index, yielding a sensitivity of 56.3% and a specificity of 92.3%. It had a positive predictive value of 69.2% and negative predictive value of 87.3%. The SI-Bord had adequate discriminative power between cases and non-cases of BPD, with the area under the ROC curve being 0.83. Cronbach’s alpha for the SI-Bord was 0.76, indicating acceptable internal consistency. The SI-Bord score was positively correlated to PHQ-9 and PSS-10 scores (*r* = 0.67 and *r* = 0.69, *p* < 0.001, respectively) and negatively correlated to MSPSS (*r* = − 0.50, *p* < 0.001). The prevalence of BPD in the sample was 6.4%, according to the cut-off score > 9.

**Conclusion:**

The SI-Bord demonstrated good reliability and validity for screening BPD in university students. However, a study in non-Thai and other population groups should be warranted.

## Background

As a group, university students commonly experience stress and depression as they transition from adolescence to early adulthood, which often requires them to adapt to a new social role and identity, to maintain interpersonal relationships, to manage their own finances, and to strive for academic success [1]. People during this period of life are more likely to face challenges such as emotional dysregulation, maladaptive behaviour, poor impulse control, drug or substance abuse, and even self-harm [2]. Studies verify that university students have high levels of stress [3, 4] and a systematic review reported that the prevalence of depression among university students ranged from 10 to 85% (with a weighted mean prevalence of 30.6%) [5]. In addition to depression, a US study found that 24% of the undergraduate sample had suicidal ideation and 9% had attempted suicide [6]. In comparison, the lifetime prevalence of suicidal ideation in adults worldwide is 9%, and the prevalence of suicide attempts is 2.7% [7].

One important factor associated with suicidality is borderline personality disorder (BPD), which usually become apparent during adolescence and young adulthood [8, 9]. According to the *Diagnostic and Statistical Manual of Mental Disorders*, fifth edition (*DSM-5*), BPD is characterized by ‘a pervasive pattern of instability of interpersonal relationships, self-image, and affects, and marked impulsivity beginning by early adulthood and present in a variety of contexts’ [10]. BPD is closely related to depression [11–13] and the unstable moods, impulsivity and violent behaviour characteristic of BPD make it highly correlated with suicide attempts [14]. BPD is also associated with poor social support [15].

As for the prevalence of BPD among university students, a review of 43 studies reported that the prevalence of BPD ranged from 0.5 to 32.1%, with an unadjusted lifetime prevalence of 9.7% [16]. It is, therefore, important to detect BPD early to give at-risk individuals the opportunity to receive treatment promptly to prevent further complications and comorbidities.

Up to 13 measurements are used to diagnose BPD [16] in widely-used screening tools such as the McLean Screening Instrument for BPD (MSI-BPD) [17], the Borderline Personality Questionnaire (BPQ) [18], the PDQ-4 BPD [19], the International Personality Disorder Examination (IPDE) [20], and the Structured Clinical Interview for DSM-IV Axis II Personality Disorders-Patient Questionnaire – BPD Scale (SCID-II-PQ BPD) [21]. These measurements use either self-reported data or clinician ratings. The number of items on these scales ranges from 9 to 80 items. When comparing four different scales, Chanen and colleagues [22] found that the longer scales (BPQ (80 items) and SCID-II PQ (15 items)) had better overall diagnostic accuracy (with the area under the Receiver Operating Characteristics curve (AUC) = 0.80 for SCID-II PDQ and AUC = 0.91 for BPQ), while the shorter scales (MSI (10 items) and IPDE-BPD (9 items)) provided poorer AUC measurements of 0.73 and 0.77, respectively. Likewise, van Alebeek and colleagues [23] compared three scales (MSI (10 items), PDQ (10-items), and SCID-II-PQ BPD (15 items)) in adolescents and young adults and found that all instruments predicted a BPD diagnosis equally well, despite the fact that PDQ’s internal consistency was quite poor. In addition, studies have shown that borderline personality feature especially emotion dysregulation, the most sensitive and specific manifestation for BPD diagnosis [24], are influenced and can be shaped by culture [25–27].

Recently, the authors (NW, TW, and PK) developed a short screening tool for borderline personality disorder, comparable to the BPD subscale in SCID-II (Short-Bord) that uses a true-false questionnaire with five items based on *DSM-5* criteria. Short-Bord was tested in psychiatric patients who came for psychotherapy and showed an AUC of 0.95, a sensitivity of 91.2% and a specificity of 85.4%, determined by a cut-off of ≥2, and a Cronbach’s alpha of 0.80 [28]. Although the scale is brief, it performs well and has promise as a diagnostic tool. However, Short-Bord has never been tested in a non-clinical sample, especially not one of university students. In this study, we would like to extrapolate the test to this population. For this setting, the ideal diagnostic instrument must be short while diagnosing BPD as accurately as longer tools.

This version – the short instrument for borderline personality disorder (SI-Bord) – is, therefore, an improved version of Short-Bord, which was reliable and valid. Compared with other current screening instruments, the SI-Bord is shorter and culturally validated (Additional file 1: Appendix). The objectives of this study were to administer the SI-Bord to a sample of university students to 1) test the diagnostic validity of the SI-Bord, and 2) test its reliability and validity compared to other psychological measurements.

## Methods

This study used a cross-sectional online survey of 342 undergraduate students in Thailand between November and December 2019. To participate in the online survey, the participant had to read the protocol and accept an informed consent document on the first page of the questionnaire. Participants who objected to the informed consent document were directed to the end of the survey. No respondent was forced to participate and he/she could withdraw at any time. For the interview and video recording of the interview, both the interviewer and the participant were informed that the proceedings would be private and confidential. The study was approved by the Ethics Committee of the Faculty of Medicine at Chiang Mai University, Thailand.

### Participants

The participants were undergraduate students located all over Thailand, aged18–25, who spoke Thai. The exclusion criteria included being diagnosed with schizophrenia, bipolar disorder, drug or alcohol use disorder, and being intoxicated with alcohol within 24 h prior to participating in the study. Participants were asked to complete the questionnaires on the Internet via personal computers, laptops, smartphones, or tablets.

Those who scored ≥1 on the Short-Bord were randomly selected to participate in a long-distance interview (using Zoom video conferencing) by one of the psychiatrist investigators (NK, AO, NW, and TW) to confirm the diagnosis using the Structured Clinical Interview for DSM-IV Axis II PDs (SCID II). Video of the interviews was recorded for later review.

Sample size estimation was calculated for a prevalence study, which the estimated prevalence of BPD in the university sample from previous study was 30%, the precision was set at 5%, and the confidence level was 0.95. This yielded an estimated sample size of 323. The dropout rate was determined to be 10%. The estimated total sample was 355 for the survey.

**Fig. 1:**
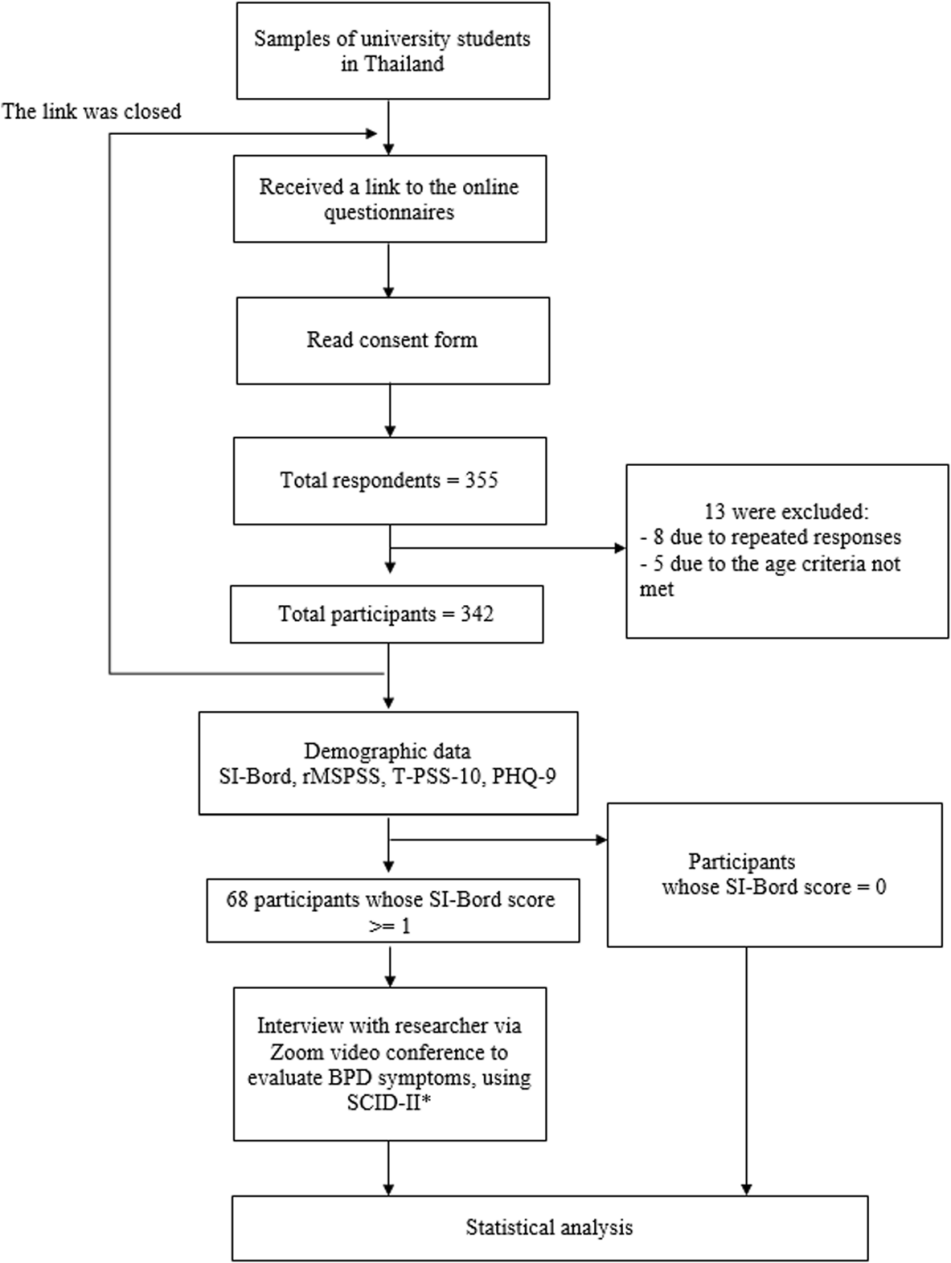
Flow chart of the study. SI-Bord = Screening instrument for borderline personality disorder, r-MSPSS = Revised Thai Multi-Dimensional Scales of Perceived Social Support, TPSS = Thai version of perceived stress scale, PHQ-9 = Patient health questionnaire-9

To calculate a sample size for ROC analysis, we expected an AUC of 0.725 for the SI-Bord, which was significant compared to the null hypothesis value 0.5 (no discriminating power). We expected to include thrice as many negative cases as positive cases based on the prevalence. An α-level was set to 0.05, and a β-level was set to 0.20 (power is 80%). This yielded a sample size of 68 (17 for positive cases, and 51 for negative cases) [29]. (See Fig. 1).

### Inter-rater reliability

Four psychiatrist investigators, with 2, 4, 18 and 28 years of working experience, administered the SCID-II BPD for each interview. None of them knew the participants they had to interview. They were randomized to interview the respective participant arranged by research assistants. Before conducting an interview, an inter-rater agreement was examined using joint video. We had ten cases (mixed both borderline and nonborderline patients) in the process of interrater reliability. All ten cases were interviewed by four psychiatrists. Two psychiatrists interviewed two cases each, and three psychiatrists interviewed three cases each. Each case was interviewed by one interviewer, and the other three psychiatrists rated the symptoms independently by watching the videos. All four psychiatrists were blinded to all cases. This totaled six pairs of interviewers for each video, and 60 pairs for all comparisons. No SCID-II Personality Questionnaire was used before any interview. The intraclass correlation analysis yielded a coefficient of 0.925, which was considered excellent.

### Instruments

In addition to the sociodemographic data, e.g., age, sex, academic year, income, etc., the participants were asked to complete the following measurements.

1. **Screening instrument for borderline personality disorder (SI-Bord)**

SI-Bord was modified from the Short-Bord [28], consisting of 5 questions representing the DSM-5 criteria of BPD for 1) abandonment avoidance, 2) interpersonal relationships instability, 3) identity disturbance, 4) suicidal and self-harm behaviors and 5) affective instability. SI-Bord has 4-point Likert scales, ranging from never (0) to very often (3), while the original version of the Short-Bord uses a true-false response. This extended response category was intended to increase reliability [30]. In addition, the content of each item was also modified, be more understandable, based on Thai cultural context. For example, item 3 (identity disturbance) read “My feelings suddenly changed, such as “I don’t know who I am,” “I don’t know where I am going“ or “I feel lonely deep down” or “I have no goal in life” The total score ranges from 0 to 15. The higher score represents more BPD symptoms or traits. Si-Bord takes 1–2 min to complete. The study sample yielded a Cronbach’s alpha of 0.76.

2) **Revised Thai Multi-Dimensional Scales of Perceived Social Support (r-MSPSS)**

This tool measures the extent to which an individual has experienced being support by significant others (SO), friends (FR), and family (FA) [31]. It has 12 questions with 7-point Likert scales ranging from very strongly disagree (0) to very strongly agree (6). The higher the score, the higher the level is attained of perceived social support. The revised-Thai version demonstrated good psychometric properties [32]. The study sample yielded a Cronbach’s alpha of 0.91.

3) **Thai version of Perceived Stress Scales (T-PSS-10)**

The T-PSS-10 is a 10-item self-reporting questionnaire measuring the extent to which an individual perceived stress over the past 4 weeks. It uses a 5-point Likert scale ranging from never (0) to very often (4). The higher the total score, the higher the level is attained of feeling stress. T-PSS-10 demonstrates good psychometric properties [33]. The study sample yielded a Cronbach’s alpha of 0.85.

4) **Patient-Health Questionaire-9 (PHQ-9)**

The PHQ-9 is a 9-item self-reporting questionnaire measuring the extent to which an individual has experienced depressive symptoms over the past 2 weeks [34]. The 4-response Likert scale ranges from 0 (not at all) to 3 (nearly every day). The higher the total score, the higher the level is attained of depressive symptoms. The Thai version PHQ-9 showed a Cronbach’s alpha of 0.79 and a significant association between the PHQ-9 and the HAM-D [35]. The study sample yielded a Cronbach’s alpha of 0.89.

5) **Thai version of Structured Clinical Interview for DSM-IV Axis II PDs (T-SCID II)**

T-SCID-II is a structured clinical interview for personality disorder that has been used to diagnose personality disorders based on DSM-IV and DSM-IV TR [36]. The Thai version demonstrated a promising interrater reliability of ≥0.8 [37, 38]. In this study, only the BPD section was used.

### Statistical analysis

For sociodemographics and scores of psychological measurement, descriptive statistics, e.g., frequency, percentage, mean and SD were used. In evaluating the diagnostic validity of the SI-Bord, receiver-operating characteristics (ROC) analyses were used to calculate the values of sensitivity, specificity, positive predictive value (PPV) and negative predictive value (NPV). We calculated the Youden index ***J*** [39] which is defined as: J = max (sensitivity_*c*_ + specificity_*c*_ - 1), where c ranges over all possible criterion values. MedCalc Statistical Software, Version 19.2.1 (MedCalc Software Ltd., Ostend, Belgium) was used for ROC analysis. MedCalc was used to calculate the optimal criterion and associated sensitivity and specificity, and the optimal criterion value which takes into account not only sensitivity and specificity, but also disease prevalence, and costs of various decisions. Cost, as defined by MedCalc, is the average cost resulting from the use of the diagnostic test at that decision level such as the cost of doing the test, which is constant at all decision levels. ROC analyses were also used for the area under the curve calculation (AUC) to determine the performance of the test.

Interrater reliability calculated by intra-class correlation coefficient (ICC) was used for rater’s agreement. An ICC value of > 0.8 was acceptable. Internal consistency of the instrument was determined by Cronbach’s alpha, for which a value of > 0.7 was considered acceptable. Concurrent validity of the SI-Bord was examined using Pearson’s correlation coefficients between SI-Bord and with other measurements, i.e., rMSPSS, T-PSS-10, and PHQ-9. All analyses, except for ROC, were performed using IBM SPSS, Version 22.

## Results

The total number of responses was 355. Among them, 13 were excluded: five respondents were older than the inclusion criteria (25 years old) and eight were repeat responses. The final number of participants was 342, with 275 females (80.4%) and a mean age of 20.25 (SD = 1.4). The majority of the participants were health science students. Among the 68 participants who were interviewed, 45 were females and 23 were males. Sixteen (11 female and 5 male) participants met the criteria for BPD. Other characteristics are shown in Table 1.

**Table 1:** Characteristics and demographic data of the participants (*N* = 342)

Variables		N	%
Sex	Female	275	80.41
Years of Studying	1	90	26.32
	2	78	22.81
	3	93	27.19
	4 or more	81	23.68
Marital Status	Single	341	99.71
	Divorced	1	0.29
Faculty classification	Health Science	281	82.16
	Technology	27	7.89
	Social Science	28	8.19
	Unspecified	6	1.75
Monthly Allowance (Baht)	< 4500	106	30.99
	4500–6000	126	36.84
	> 6000	110	32.16
Satisfaction of monthly allowance	Yes	262	76.61
	No	80	23.39
Part-time job	Yes	59	17.25
	No	243	71.05
**Mean scores of Psychological measures**		**Mean**	**SD**
SI-Bord		3.80	2.97
r-MSPSS		53.09	12.76
-SO		16.08	6.05
-FA		19.27	4.94
-FR		17.74	4.80
T-PSS		14.74	6.55
PHQ-9		6.27	5.33
**SCID-II (*N*** **= 68)**			
Met criteria for Borderline personality disorder			
Yes		16	23.5
No		52	76.5

*r-MSPSS* revised Thai Multidimensional Scale of Perceived Social Support, *SO* Significant Others, *FA* Family, and *FR* Friends

**Fig. 2:**
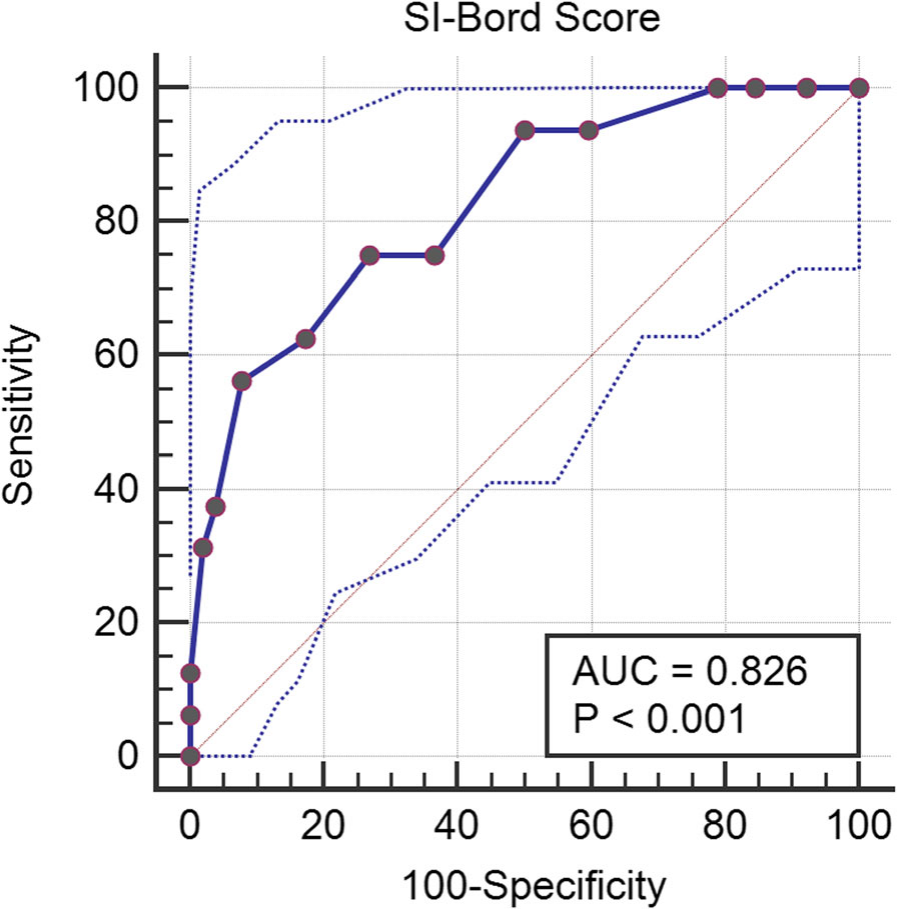
ROC curve with 95% Confidence Inerval for the SI-Bord. dotted lines = upper and lower confidence limit of the SI-Bord, bold line = SI-Bord score, AUC = area under the curve

The ROC curve was analyzed to determine the cut-off score of the SI-Bord (Fig. 2). The AUC was 0.826 (95% CI, 0.715–0.907; *p* < 0.0001), suggesting an 83% chance that the SI-Bord will correctly distinguish a BPD case from a non-BPD case. Table 2 shows the sensitivity and specificity according to different cut-off scores of the Short-Bord against the diagnosis made by psychiatrists using SCID-II. Youden’s index was 0.4856, using the cut-off > 9, which yielded a sensitivity of 56.25% and a specificity of 92.31%. Optimal criterion, which accounted for disease prevalence (23.5%) and estimated costs, was > 9. Table 2 shows that Youden’s index and optimal criterion suggested a cut-off of > 9, which yielded a sensitivity of 56.25% (95%CI, 29.9–80.2%), a specificity of 92.31% (95%CI, 81.5–97.9%), a positive predictive value of 69.2% (95%CI, 44.4–86.4%), a negative predictive value of 87.3% (95%CI, 79.6–92.3%), and a cost of 0.162 units.

**Table 2:** Sensitivity and specificity using each cut-off score (*N* = 68)

Criterion	Sensitivity	95% CI	Specificity	95% CI	+PV	95% CI	-PV	95% CI	Cost^a^
≥1	100.00	79.4–100.0	0.00	0.0–6.8	23.5	23.5–23.5			0.765
> 1	100.00	79.4–100.0	7.69	2.1–18.5	25.0	23.6–26.5	100.0		0.706
> 2	100.00	79.4–100.0	15.38	6.9–28.1	26.7	24.5–29.0	100.0		0.647
> 3	100.00	79.4–100.0	21.15	11.1–34.7	28.1	25.3–31.0	100.0		0.603
> 4	93.75	69.8–99.8	40.38	27.0–54.9	32.6	27.2–38.5	95.5	75.4–99.3	0.471
> 5	93.75	69.8–99.8	50.00	35.8–64.2	36.6	29.9–43.8	96.3	79.3–99.4	0.397
> 6	75.00	47.6–92.7	63.46	49.0–76.4	38.7	28.6–49.9	89.2	77.5–95.2	0.338
> 7	75.00	47.6–92.7	73.08	59.0–84.4	46.2	33.5–59.3	90.5	80.0–95.8	0.265
> 8	62.50	35.4–84.8	82.69	69.7–91.8	52.6	35.4–69.2	87.8	79.0–93.2	0.221
>9^b,c^	56.25	29.9–80.2	92.31	81.5–97.9	69.2	44.4–86.4	87.3	79.6–92.3	0.162
> 10	37.50	15.2–64.6	96.15	86.8–99.5	75.0	40.1–93.1	83.3	77.3–88.0	0.176
> 11	31.25	11.0–58.7	98.08	89.7–100.0	83.3	38.6–97.5	82.3	76.9–86.6	0.176
> 12	12.50	1.6–38.3	100.00	93.2–100.0	100.0		78.8	75.5–81.7	0.206
> 13	6.25	0.2–30.2	100.00	93.2–100.0	100.0		77.6	75.3–79.7	0.221
> 14	0.00	0.0–20.6	100.00	93.2–100.0			76.5	76.5–76.5	0.235

*+PV* positive predictive value, −*PV* negative predictive value, *CI* confidence interval, ^a^Taking into account disease prevalence (23.5%) and estimated costs, ^b^Youden index J, ^c^Optimal criterion

Table 3 shows that all SI-Bord items had a significant positive correlation with each other and with the total score. Item 1 (avoiding abandonment) seems to have the weakest correlation with the total score (*r* = 0.598, *p* < 0.001), while item 2 (unstable relationship) had a nonsignificant correlation with the psychiatrists’ diagnoses (*r* = 0.071, *p* = 0.564). Factor analysis results show that a one-factor solution model fits the data best. The total variance explained by all items was 51.42% with an eigenvalue of 2.57. The factor loading coefficient ranged from 0.548 (item 1) to 0.832 (item 5), consistent with the strength of the item-total correlation coefficient, which ranged from 0.376 (item 1) to 0.660 (item 5).

**Table 3:** Factor analysis results of SI-Bord, Correlation between SI-Bord items and Correlation with the diagnoses made by psychiatrists

SI-Bord Item	1	2	3	4	5	Item-total correlation	Diagnosis	Factor loading
1. Avoid abandonment	1					.376^**^	.323^*^	.548
2.Unstable relationship	.302^**^	1				.471^**^	.071	.653
3.Identity disturbance	.299^**^	.424^**^	1			.646^**^	.393^*^	.823
4.Self-harm	.214^**^	.261^**^	.464^**^	1		.486^**^	.336^*^	.689
5.Mood change	.325^**^	.400^**^	.640^**^	.495^**^	1	.660^**^	.559^**^	.832

*p<.01, **p<.001, *SI-Bord* Screening instrument for borderline personality disorder

Table 4 shows the correlation between the SI-Bord and other psychological measurements. As expected, the SI-Bord was positively correlated with depression and perceived stress (*r* = 0.669 to *r* = 0.690, *p* < 0.001) but was negatively correlated with perceived social support (*r* = − 0.520, *p* < 0.001).

**Table 4:** Correlation among SI-Bord, r-MSPSS, PSS, and PHQ-9 (*N* = 342)

	rMSPSS - SO	rMSPSS- FA	rMSPSS- FR	rMSPSS- Total	TPSS-10	PHQ-9
rMSPSS- FA	.406^**^					
rMSPSS- FR	.463^**^	.583^**^				
rMSPSS- Total	.805^**^	.799^**^	.821^**^			
TPSS	−.249^**^	−.468^**^	−.423^**^	−.458^**^		
PHQ-9	−.313^**^	−.501^**^	−.473^**^	−.520^**^	.722^**^	
SI-Bord	−.301^**^	−.494^**^	−.447^**^	−.502^**^	.669^**^	.690^**^

**P<.001, *r-MSPSS* revised Thai Multidimensional Scale of Perceived Social Support, *SO* Significant Others, *FA* Family, and *FR* Friends, *TPSS* Thai version of perceived stress scale, *PHQ-9* Patient health questionnaire-9, *SI-Bord* Screening instrument for borderline personality disorder

## Discussion

The aim of this study was to test the efficacy of the SI-Bord on university students, and the results showed it is both valid and reliable for this population. Most importantly, the results indicate that the SI-Bord is satisfactory in its diagnostic accuracy, which is comparable to other vetted screening questionnaires such as the BPQ (AUC = 0.83) and the BDQ (AUC = 0.85) [22], despite having fewer items. The Cronbach’s alpha of the SI-Bord, while not excellent, is acceptable.

The SI-Bord’s AUC of 0.83 means that a randomly selected individual from the positive group will have a test value larger than that of a randomly chosen individual from the negative group 83% of the time [40]. Compared to other relatively short instruments such as the MSI and IPDE, the SI-Bord is even more succinct, and is even more predictive.

Unlike MSI or IPDE, the SI-Bord uses only the most important diagnostic criteria of BPD instead of using all 9 *DSM* criteria. As a screening tool, only those items that correlate strongly with the reference diagnosis were counted [28, 41]. It is easier to form a unidimensional scale with only a few items, making it logical to use both the total score and cut-off score. Related studies revealed that some brief screening tools, such as MSI, had multi-factor solution models [42, 43]. Needless to say, the lengthier scales also had more than one factor [44, 45], rendering the total score less legitimate because of its multidimensional construct [46].

Despite its unidimensional scale, item 2 (unstable relationship) on the SI-Bord was not productive for the scale as it could not discriminate between cases and non-cases. It had a nonsignificant correlation with the psychiatrists’ diagnoses despite being uniformly consistent with the other four items as indicated in previous research [47]. This reduced the overall accuracy of the scale. The scale’s predictive accuracy would be higher if this item had functioned as intended. This item may not have been diagnostically useful because most of the participants were not in romantic relationships. It is also possible that respondents were confused about what kind of relationship this item referred to and, hence, this item may not provide useful information to the scale compared to other items. Moreover, it might be possible however that this BPD dimension be unstable and difficult to capture [48]. Having this item revised or replaced with another significant item may improve the diagnostic accuracy of the SI-Bord.

As a screening tool, the SI-Bord gives clinicians the option of using various cut-off scores depending on the clinical purpose. For example, to increase the probability of including a case, a cut-off score of > 7 has higher sensitivity (75.0%) than the cut-off of > 9 (56.25%) but using the lower cut-off score could increase the chance of a false positive. The negative predictive value differed little between the two scores (90.5% vs 87.3%) but the positive predictive value differed greatly (46.2% vs 69.2%). Therefore, a cut-off of > 9 would be preferred over the cut-off > 7 when researchers are interested in excluding non-cases from the study [49]. In addition, some investigators take prevalence and costs into account when calculating the optimal cut-off score [40]. Using the current prevalence of 6.4%, MedCalc suggested a cut-off > 9, as it yielded the lowest cost of 0.162 units.

Our study indicates that the validity of the SI-Bord is similar to other psychological measurements and one interpretation of our results is that a high score on the SI-Bord, is, as expected, associated with a higher level of depression and perceived stress and a lower level of perceived social support, which is consistent with other related studies [11, 50]. This underscores the importance of detecting BPD early. This screening process should be conducted proactively in a convenient and practical manner to allow students to receive appropriate and timely interventions before the experience of stress, depression and lack of social support escalates to suicidality. Future research is to correlate the diagnostic accuracy of the SI-Bord and latest functional near infrared spectroscopy which could distinguish depressive disorder and borderline personality disorder [51].

### Strengths and limitations

The SI-Bord is, to the best of our knowledge, the first screening tool for BPD specifically designed to screen university students. The SI-Bord is a unidimensional scale that, although short, is comparable with longer scales in terms of its psychometric properties and its ability to distinguish between participants with and without BPD.

This study has some limitations. The information obtained in the interviews using the SCID-II diagnosis was limited, as it was obtained only from the participants and only used participants’ self-reported history to exclude those with clinical disorders (e.g. bipolar disorder, schizophrenia). The accuracy of the data may have been impacted by a lack of corroborating data. Furthermore, the data in this study were obtained from participants with access to computers and other smart devices. Those who were excluded because they were unable to access the survey may also be at risk for BPD. This study did not exclude other common comorbidity in Asia such as Internet addiction that was found to be common in university students [52] and adolescents [53]. Finally, the participants were in their late adolescence or early adulthood and may experience mood swings, which would have been difficult to interpret in some cases.

## Conclusion

The SI-Bord showed good validity and reliability in screening for BPD. It is a brief and reliable screening tool for university students. Further investigation should be encouraged, especially to determine which items yield the most accurate and reliable results.

## Supplementary information

**Additional file 1: Appendix.** Screening instrument for borderline personality disorder (SI-Bord).

## Data Availability

The datasets used and/or analyzed during the current study are available from the corresponding author on reasonable request.

## Abbreviations

AUC: Area Under the Receiver Operating Characteristics Curve
BPD: Borderline Personality Disorder
BPQ: Borderline Personality Questionnaire
DSM-5: Diagnostic and Statistical Manual of Mental Disorders, fifth edition
ICC: Intraclass Correlation Coefficient
IPDE: International Personality Disorder Examination,
MSI: McLean Screening Instrument for BPD
PHQ-9: Patient-Health Questionaire-9
r-MSPSS: Revised Thai Multi-Dimensional Scales of Perceived Social Support
ROC: Receiver-Operating Characteristics
SCID-II-PQ: Structured Clinical Interview for DSM-IV Axis II Personality Disorders-Patient Questionnaire
Short-Bord: Short screening tool for borderline personality disorder
SI-Bord: Short instrument for borderline personality disorder
T-PSS-10: Thai version of Perceived Stress Scales
