# Supplementary material for: Development and validation of a screening instrument for borderline personality disorder (SI-Bord) for use among university students
Source: BMC Psychiatry. 2020 Aug 17;20:479. doi: 10.1186/s12888-020-02807-6 (PMC7526163; doi:10.1186/s12888-020-02807-6)
Supplement: Supplementary file 1 — Additional file 1: Appendix. Screening instrument for borderline personality disorder (SI-Bord). [file 12888_2020_2807_MOESM1_ESM.docx]

**Appendix:** **Screening instrument for borderline personality disorder (SI-Bord)**

| How much of the following emotions and characteristics match the characteristics of a person, please mark √ in the box on the right. | Not at all | A little | somewhat | To a great extent |
| --- | --- | --- | --- | --- |
| 1. When people with ties to me leave me, I can barely live. |  |  |  |  |
| 1. The relationship between me and those I am bound to fluctuate between when good is very good and when bad is very bad. |  |  |  |  |
| 1. My feelings suddenly change, such as "I don't know who I am," "I don't know where I am going" or "I feel lonely”, “I have no goals". |  |  |  |  |
| 1. I threaten to hurt myself or attempt to hurt myself or have attempted suicide. |  |  |  |  |
| 1. My mood changes suddenly, for example, from normal to irritability, depression, or anxiety. |  |  |  |  |
